# Recent Advances of Polyphenol Oxidases in Plants

**DOI:** 10.3390/molecules28052158

**Published:** 2023-02-25

**Authors:** Song Zhang

**Affiliations:** College of Food Science and Engineering, Shandong Agricultural University, 61 Daizong Street, Tai’an 271018, China; zhangsong5861927@sdau.edu.cn

**Keywords:** polyphenol oxidase, biological function, plants, recent advances

## Abstract

Polyphenol oxidase (PPO) is present in most higher plants, but also in animals and fungi. PPO in plants had been summarized several years ago. However, recent advances in studies of PPO in plants are lacking. This review concludes new researches on PPO distribution, structure, molecular weights, optimal temperature, pH, and substrates. And, the transformation of PPO from latent to active state was also discussed. This state shift is a vital reason for elevating PPO activity, but the activation mechanism in plants has not been elucidated. PPO has an important role in plant stress resistance and physiological metabolism. However, the enzymatic browning reaction induced by PPO is a major problem in the production, processing, and storage of fruits and vegetables. Meanwhile, we summarized various new methods that had been invented to decrease enzymatic browning by inhibiting PPO activity. In addition, our manuscript included information on several important biological functions and the transcriptional regulation of PPO in plants. Furthermore, we also prospect some future research areas of PPO and hope they will be useful for future research in plants.

## 1. Introduction

Polyphenol oxidase (PPO) is a copper-containing phenolase discovered in most animals, plants (not found in *Arabidopsis thaliana*, *Brassica napus,* and green algae), and microorganisms [1,2,3]. In plants, PPO is encoded by multiple genes from the nuclear genome. Various PPO genes are found in the same plant. Nine PPO genes (*StPOTP1*, *StPOTP2*, *StPOT32*, *StPOT33*, *StPOT72,* and *StuPPO5*–*StuPPO9*) were isolated in potato [4,5,6]. Also, four PPO genes (*BPO1*, *BPO11*, *BPO34,* and *BPO35*) were cloned in bananas [7]. Tomato contains seven PPO genes, whereas just one PPO gene was found in cucumber [8,9]. The largest number of PPO genes exists in *Salvia miltiorrhiza,* which contains 26 members [10].

**Figure 1:**
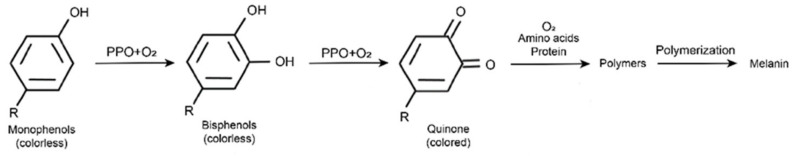
PPO catalyzes monophenol hydroxylated to *o*-diphenol and catalyzes *o*-diphenol to *o*-quinone. Then, *o*-quinone further polymerizes and condenses with amino acids and proteins to produce brown substances.

The biological and chemical properties of PPO have been studied for more than a century, since 1896 [11]. Most of the PPO in plants is found in the plastids, such as the chloroplasts of photosynthetic cells and the leucoplasts of storage cells, etc., and is relatively more abundant in young tissues. Based on substrate specificity and action mode, scientists usually divide PPO into tyrosinase (EC 1.14.18.1), catechol oxidase (EC 1.10.3.1), and laccase (EC 1.10.3.2) [12,13]. PPO catalyzed two important reactions: the monophenol is hydroxylated to *o*-diphenol, and *o*-diphenol is oxidized to *o*-quinone [14]. Then, *o*-quinone was further polymerized and condensed with amino acids and proteins to produce brown substances (Figure 1) [15,16]. The activity of PPO conforms to the parabolic law at different pHs, with the highest activity and the strongest catalytic activity at the optimum pH. Multiple optimum pH of PPO had been confirmed in plants depends on its substrate and original species. Furthermore, the pH optimum of most PPO was range from 5.0 to 8.0. Besides pH optima, temperature also plays a vital role in PPO activity. PPOs in different plant species showed varied optimum temperatures; most of them were in the range of 30–50 °C. PPO with the highest catalytic activity at the optimum pH and temperature [2,14].

During the postharvest process, the enzymatic browning involved in PPO is usually unfavorable, particularly manifested in deteriorated appearance and decreased nutrition [17]. According to statistics, browning caused about 50 percent of the loss of fruits and vegetables during processing [18]. Therefore, lots of research has been focused on how to inhibit the activity of PPO [19]. Currently, a number of new methods have been reported to decrease the browning in purple sweet potatoes, pears, potatoes, loquats, and other crops [16,20,21,22]. PPO plays an important role in plant physiology, except for enzymatic browning. According to previous reports, PPO was crucial for plants to resist microorganisms and herbivorous insects [23]. Tomatoes with high expression of PPO showed significantly increased disease resistance to *P. syringae* [24]. In addition, the researchers found that PPO played a positive role in enhancing resistance to both cotton bollworm and beet armyworm in tomato [25]. Felton et al. displayed that the activity of PPO was negatively correlated with the fruit worms [26].

Jukanti and Aravind reviewed some early research progress and insightful reflections in the book “Polyphenol Oxidases (PPOs) in Plants” in great detail [27]. And, some aspects of PPO, including enzymatic features, substrate specificity, transcriptional and post-transcriptional regulation and physiological roles have been discussed in several articles [19,28,29]. However, a comprehensive summary of the current research advances on understanding polyphenol oxidases (PPOs) in plants is missing. In this manuscript, we summarize various classic manuscripts and the latest research aspects of PPO in recent years. The latest research on the physicochemical properties, functions, and regulation of PPO (pH, temperature, substrate specificity, molecular weight, enzymatic browning, physiological functions, etc.) is listed. In addition, we propose better suggestions for the study of the mechanism of action of PPO, hoping they will be useful for future research.

## 2. Distribution and Functional Domain of PPO

PPO was synthesized in the ribosome and entered the plastid in the form of a zymogen in an inactive (latent) state [30]. Arnon found that PPO is present in the chloroplasts of *Beta vulgaris* [31]. Murata et al. confirmed that the subcellular localization of apple’s PPO is in plastids and chloroplasts [32]. Onsa et al. observed that the PPO of *Metroxylon sagu* exists in the amyloplast, mitochondria, endoplasmic reticulum, and Golgi complex using an electron microscope [33]. Now, it is generally believed that PPO can be free in the cytoplasm and in thylakoids or other non-green plastid vesicles in plants [34,35]. Moreover, the contents of PPO vary widely in both temporal and spatial dimensions within the same plant. PPO contents were higher in young tissues and lower in mature and senescent tissues, showing temporal differences [10]. And spatial differences were manifested as large variations in PPO content and types in different tissues during the same growth period. For example, PPO in tubers is mainly encoded by the *StPOT32* gene, whereas in roots, PPO is derived from *StPOT72* in potato [5]. At the same period, the PPO activity in olive fruits was significantly higher in cotyledons than in leaves. And PPO protein levels increased significantly during the olive ripening process [36].

PPO contains three important domains: an amino-terminal (N-terminal) domain, a highly conserved type-III copper center, and a carbon-terminal (C-terminal) region [12,37,38,39]. Its N-terminal contains a plastid transit signal peptide that can target the membrane of plastids. With the signal sequence cleaved by signal peptidase, the mature PPO (identified as the PPO latent state) was located in the plastid [40]. Therefore, the main function of the N-terminal domain is to mediate the conversion of precursor PPO into plastids. The catalytically active region has a binuclear copper center with two copper ions (CuA and CuB). CuA is involved in the solubilization of PPO in water, whereas CuB is linked to the substrate. Furthermore, an imidazole nitrogen ligand binds 2 copper ions to 6 or 7 histidine residues, resulting in a specific three-dimensional active site [2,13]. In a small number of plants, PPO contains a trinuclear copper center (CuA, CuB, and CuC), and CuC is the linkage site for molecular oxygen [4,41]. The C-terminal domain is related to the activation of latent PPO. It plays an important role in shielding the active site of the copper center. Activated PPO with catalytic activity is generally considered to be cleaved from the C-terminal by protease or under stress to expose the active sites [42,43,44]. However, studies about the C-terminus, which contains large numbers of protease recognition sites but is poorly stable, are deficient [45].

## 3. Optimal Conditions of PPO

The relevant physicochemical properties of PPO have been reported comprehensively. It is certain that PPO exhibits different characteristics in different plants and even varies in different parts of the same plant or in different growth periods.

### 3.1. Optimal pH of PPO

The activity of PPO conforms to the parabolic law at different pHs, with the highest catalytic activity at the optimum pH. Acidic and alkaline conditions will lead to a decrease in the catalytic activity of PPO [35]. It is beneficial to study the activity of PPO at different pH levels in order to control the catalytic action and understand the physicochemical properties, or chemical reactions, of PPO. Usually, the optimum pH of PPO in plants depends on its substrate and original species [46]. According to recent articles, the optimum pH of PPOs is varied in different plants (see Table 1). Moreover, the optimum pH corresponding to different substrates varies widely in the same plants. Catechol was mostly described as the substrate. We could find that the optimum pH of PPO was 7.0 for African bush mango (*Irvingia gabonensis*) fruit peel, tomato (*Solanum lycopersicum*), sweet potato (*Ipomoea batatas* L. Lam), and Areca nut (*Areca catechu* L.) kernel when catechol was served as substrate (Table 1) [16,47,48,49].

For the same substrate, the optimum pH of PPO is varied in different cultivars. *Lilium lancifolium*. Thunb, *Lilium brownie* var. *viridulum,* and *Lilium davidii* var. *unicolor* are three cultivars that show 4.0, 4.0, and 6.5–7.0 optimum pH for PPO with catechol as substrate, respectively [50]. The PPO in the same tissue of the same cultivar has a different optimal pH for different substrates. The optimum pHs of PPO in fennel (*Foeniculum vulgare* Mill.) seeds were 6.0, 5.0, 5.0, and 7.0 when catechol, 4-methylcatechol, 4-tertbutylcatechol, and pyrogallol were served as substrates, respectively [51]. It was also reported in truffles (*Terfezia arenaria*) [46]. Interestingly, the same tissue contains multiple PPOs with different optimum pHs. For example, PPO1 and PPO2 are present in tea leaf (*Camellia sinensis*) with optimum pHs of 5.5 and 6.0, respectively [54]. At the same time, the optimum pH of mostly PPO is neutral, whereas a small number is acidic (Table 1) [61]. Of course, extraction methods, the environment, and many other factors will cause some fluctuation in their optimal pH [63].

### 3.2. Optimum Temperature of PPO

Temperature is a critical factor affecting the activity of PPO. Similar to pH, the catalytic activity is highest at the optimum temperature and decreases at higher or lower temperatures. Meanwhile, PPO will be inactivated under ultra-low and high temperature conditions [35]. Several factors influence the role of temperature in enzymatic browning. For example the rate of its enzymatic browning reactions increases approximately two to three times with every 10 °C increase in temperature before reaching the optimum temperature of PPO. (2) A higher temperature destroys the three-dimensional structure of PPO and reduces its catalytic activity. (3) The concentration of dissolved oxygen, which is influenced by temperature variation, also affects the enzymatic browning rate in the reaction system [63]. We summarized the optimum temperature of PPOs from 18 different plant species under the corresponding substrate, according to recent research (Table 2).

The optimum temperature of PPO, which is participating in the reaction, is generally mild and mostly in the range of 15–50 °C. The optimum temperature of the same PPO interaction with different substrates is not invariable. It was found that the temperature at which the PPO reached its maximum activity differed depending on the substrate. The PPO of truffles showed maximum activity at 30 °C, 35 °C, 40 °C, and 45 °C when the substrates 4-methylcatechol, L-tyrosine, pyrogallol, and catechol were involved in the reaction, respectively [46]. Therefore, we believe that different PPOs have varying sensitivity to temperature, and different temperatures could affect the three-dimensional structure of PPO. We suggest that the optimum temperature of PPO may have an important correlation with the environmental temperature where the plant is located. For example, the optimum temperature for the PPO of the African bush mango (*Irvingia gabonensis*) fruit peel, which grows in the tropics, is 50 °C, while the optimum temperature for the PPO of the cold-resistant lily is 15 °C [48,50]. It is concluded that the optimal temperature varies greatly between plants and substrates.

## 4. Substrate Specificity and Molecular Weight of PPO

Enzymes have strict substrate selectivity in catalytic reactions. The diversity of PPO types in different plants determines its various phenolic substances. Meanwhile, the catalytic activity of the PPO is obviously affected by the lateral chain, the number of hydroxyl groups, and the position on the benzene ring of the phenolic substrate [64]. Broadly, the hydrophobic amino acid composition of the active sites of PPO is considerably varied, which causes substrate specificity [65]. The mechanism of the substrate specificity of PPO is still inconclusive, and there are three widely discussed hypotheses: "blocker residue," "oxidative mechanism," and "second shell residues." The "blocker residues," "oxidative residues," and "second shell residues" are located near the active site and play a role in substrate specificity [29]. Panis et al. mutated the amino acid residues (Asn240, Leu244, and Phe260) of the walnut tyrosinase site, directly causing the transformation of tyrosinase to catechol oxidase [66].

According to recent reports, we found that the substrate specialties of PPO are more favorable than diphenols and tri-phenols. For example, the relative activity of PPO in elephant foot yam was much higher than monophenols when catechol was selected as a substrate [58]. The PPO activity showed a little difference in response to different substrates in African bush mango (*Irvingia gabonensis*), but it was still evident that catechol acted as the major substrate [48]. The PPO of blueberries (*Vaccinium corymbosum* L.) exhibited the same phenomenon, showing a higher affinity for catechol [56]. PPO in the Mexican Golden Delicious apple (*Malus domestica*) also showed a higher affinity for diphenols, with the most suitable substrate being 4-methylcatechol, and showed some affinity for chlorogenic acid [57]. The PPO of pomegranate arils (*Punica granatum* L. cv. Wonderful) is more inclined to use pyrogallic acid, a kind of tri-phenol, as its main substrate [67]. The substrate specificity of PPO is manifested by its affinity for different substrates. Although each PPO has a more favored substrate, we can see that one PPO can interact with a wide range of phenolics (Table 3). It was suggested that this phenomenon may be due to the isoenzymes of PPO or the overlapping substrate binding sites on PPO.

Researchers explored the substrate preference of PPO by measuring its activity using exogenous phenols as substrates in vitro. However, this method cannot well reflect the substrate preference of PPO in plants because these exogenous phenols may not exist in plants. Derardja et al. investigated the difference in endogenous phenol content between apricots before and after browning and discovered that catechins and their dimeric derivatives were the primary substrates of apricot PPO [68]. This is different from the results of their previous study in vitro (adding exogenous phenols), which were chlorogenic acid and 4-methylcatechol [55]. It is thus clear that the substrate with the highest affinity for PPO may not be the most suitable substrate in vivo. Exploring the actual endogenous substrates of PPO may be more helpful for understanding the physiological function of PPO.

PPO’s molecular weight varies between species. According to previous reports, the molecular weight of plant PPO ranges from 27 to 144 kDa, and most of them situate between 35 and 70 kDa [19,30]. In general, the molecular weight of PPO is determined by SDS-PAGE and Native-PAGE after purifying it, which exhibits a single protein band [19]. PPOs have been purified from many plants, such as kudzu, truffles, apricots, potatoes, etc. (Table 4). Teng et al. purified two PPO isozymes with different properties and molecular weights of 85 kDa and 42 kDa in tea leaves, respectively [54]. The molecular weight of PPO in kudzu is about 21 kDa, while it reaches 67 kDa in truffles and 65 kDa in plums [46,69].

## 5. Activated PPO and Enzymic Browning

### 5.1. Active and Latent States of PPO

**Figure 2:**
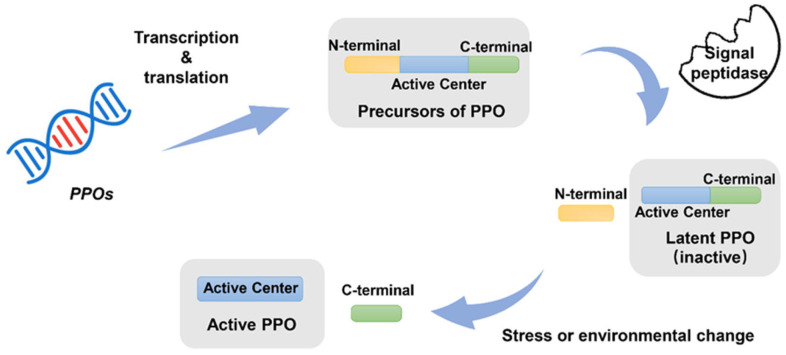
The precursor of PPO was synthesized from PPO genes. Then, the N-terminal transit peptide sequence would be removed, and a mature latent PPO (inactive) would be generated in the plastid. PPO is activated (active PPO) by cleavage of its C-terminal by a protease under stress or environmental change.

Mature PPO proteins in plants and fungi exist in both an inactive precursor state and an active state [30,71]. Mature PPO in plants is approximately 55–65 kDa, including the catalytically active region (40–45 kDa) and the C-terminal structural domain (15–19 kDa) (Figure 2) [72]. According to a previous report, the C-terminus was related to the activation of PPO. Prior to activation, PPO’s catalytic activity is almost nonexistent. After activation, PPO transforms into an activity state and contains catalytic activity (Figure 2). PPOs with different molecular weights have also been found in some other plants, such as broad bean, grape berry, sago palm, S. oleracea, sweet potato, and potato [72]. The variation in molecular weight of PPO in the same plant may also be associated with the hydrolysis of the C-terminus, which results in the active state.

It had been reported that mature PPO transformed from a latent state to an active state with the hydrolysis of its C-terminal domain in apples [55,73]. In sweet potatoes, there are two molecular weights of PPOs, 40 and 60 kDa, respectively. The 40 kDa PPO with high activity is converted from the 60 kDa form, which shows low activity. Serine protease inhibitors could completely inhibit the conversion of PPO from 60 kDa to 40 kDa in the culture of sweet potato tissue cells. This research illustrated that the activation of PPO requires the participation of proteases [74]. Derardja et al. purified latent PPO (63 kDa) and active PPO (38 kDa) and identified their sequences from apricot. They found that the molecular weight of the activated state PPO exactly matched the PPO active center region (from Asp102 to Leu429) in apricot [55].

Although there are many studies on PPO activation, most of the related reports were based on in vitro experiments to speculate on the activation mechanism in plant cells. Therefore, the mechanism of how PPO is activated in the plant is not absolutely clear. PPO’s ability to trigger enzymatic browning requires the binding of its active site to a phenolic substrate. The activation of the latent state PPO is thought to occur in spatial structure transformation [71]. Proteases, acidic environments, fatty acids, and detergents could activate the latent state of PPO. In Winters’ report, it was demonstrated that the latent state PPO can be activated by endogenous PPO substrates [72]. However, the authors hypothesized that this activation is the same as SDS activation, both of which change the steric structure of PPO. Derardja et al. obtained the latent state PPO of apricots by purification; however, the purified latent PPO was activated spontaneously along with a decrease in molecular weight [55]. Unfortunately, the reason for the spontaneous activation of latent PPO remains unclear.

### 5.2. PPO in Enzymatic Browning

Browning will not be observed in healthy plant tissues. The hypothesis of regional distribution of phenols and phenolases is the most widespread theory to explain the mechanism of enzymatic browning [75]. The subcellular localization of phenols and phenolases (PPOs) is different in plants. However, this physical partition can be broken by stresses such as wounding, high temperatures, senescence, etc. [76,77]. Then, the enzymatic browning will occur when phenolics encounter catalytically active PPO, causing the browning [78].

PPO-catalyzed enzymatic browning has a great impact on the food industry. Browning of the world’s three major beverages (tea, coffee, and cocoa) is caused by PPO, and the enzymatic browning improves their flavor and color [79,80]. But more than that, PPO also caused a lot of inconvenience in the food industry. Annually, the commercial value of a large number of fruits and vegetables is seriously reduced due to enzymatic browning [73]. Therefore, developing new methods to inhibit PPO activity has become an important research field for inhibiting enzymatic browning [19].

**Figure 3:**
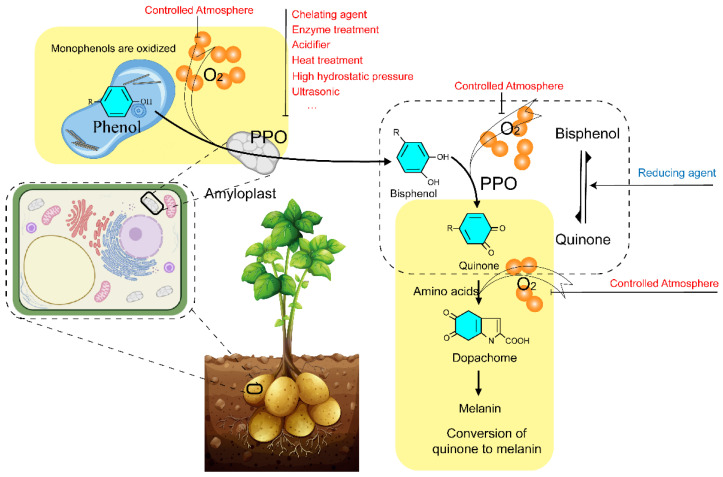
PPO in potato tubers is located in the amyloplasts, and phenols are present in the vesicles. Under oxygen conditions, the monophenols are oxidized by the activated PPO to bisphenols, which continue to be oxidized to quinones. Then quinones are converted to dopachome in the presence of amino acids and oxygen and further converted to melanin. Enzymatic browning can be inhibited by the control of oxygen, such as in a controlled atmosphere. Meanwhile, heat, enzymes, chemical treatment, etc. could decrease the biological activity of PPO and inhibit the enzymatic browning. The vector image of a potato plant was obtained from vecteezy.com. The cell pattern diagram is designed by biorender.com.

Physical, chemical, and biological methods are the most common ways to inhibit the activity of PPO (Figure 3). Physical methods such as temperature control, controlled atmosphere, high pressure, ultrasound, etc. High temperatures can rapidly deactivate PPO, but they can also negatively affect the appearance, texture, and nutritional value of plant-derived products [81]. Controlled atmosphere is widely used in fruit and vegetable production to inhibit enzymatic browning with a certain extent by increasing CO_2_ and decreasing O_2_ concentration. It is currently used in fruits and vegetables such as litchi [82], potatoes [83], lotus roots [84], apples [85], and even in graham flour [86]. High pressure treatment in water with little damage to the raw material [87] has been used in fruits such as blueberries [17] and avocados [88]. Deactivation of PPO by ultrasound is based on the physicochemical effect of the formation of tiny bubbles and cavities by self-explosion [89]. Related applications had been used in potatoes [90], blackberry juice [91], bayberry juice [92], and coffee leaves [93]. However, each method has its own advantages and disadvantages in application, so people try to use combinational methods to complement each other. For example, Xu et al. combined heat with an ultrasound method to inhibit the browning of strawberry juice [94]. Chemical methods are accomplished by adding chemicals and making use of reduction, chelation, complexation, and acidification to inhibit the activity of PPO [28]. Inhibition of enzymatic browning by chemicals is effective. It has been found that 3-mercapto-2-butanol can competitively inhibit PPO activity and effectively avoid enzymatic browning of fresh-cut potatoes [95]. Nitroprusside treatment can effectively inhibit the browning of pear juice [20]. Meanwhile, the effect of sulfite on inhibiting browning was also positive. However, safety issues have been always argued by consumers. Thus, several natural bioactive compounds have been found to be beneficial in inhibiting enzymatic browning. For example, total flavonoids isolated from young loquat fruits [96], citronella hydrosol and rose hydrosol [97], *Rosa roxburghii juice* [98], curcumin and quercetin isolated from potato [70], and endogenous phytohormone strigolactone showed inhibitory effects on PPO activity. As a natural medicine, the water extract of galla rhois can effectively inhibit the browning of apple juice [99]. In addition, biological methods that suppress PPO gene expression through antisense RNA technology are accomplished to inhibit enzymatic browning [6].

## 6. Physiological Functions of PPO

PPO is widely present in plants. And it has been reported that PPO plays important roles in plant immunity response, abiotic stresses, and physiological metabolism.

### 6.1. Response to Biotic Stresses

Plants are subject to many biotic stresses in nature, such as those caused by herbivores, insects, and microorganisms [23]. Plants generate a series of immune responses to resist the invasion. The role of PPO in plant defense mechanisms is one of several significant research fields. Previous studies have reported that PPO is related to resistance to various pathogens and insects in rice, tobacco, cotton seedlings, and apples. The earliest research on the defense of PPO against insects was through the overexpression of PPO in tomato by Felton et al. [26]. They found that the transcript levels of PPO genes were negatively correlated with the number of infested *Heliothis zea*. It was also found that the PPO could inhibit the amount of Colorado potato beetle in potatoes [100]. However, tomato PPO overexpression plants significantly reduced the growth rate and nutritional index of *Helicoverpa armigera* and *Spodoptera exigua* [25]. With the increased transcript level of PPO, the resistance to *Pseudomonas syringae* and *Alternaria solani* was also enhanced in tomato [24,101]. Furthermore, potatoes with higher PPO genes expression showed enhanced resistant to soft rots [102]. Meanwhile, the higher the PPO content, the less severe the tobacco disease [103].

Recent research suggests that the following mechanisms are involved in PPO resistance to biotic stresses [23]. (1) PPO could modify proteins by reacting with different compounds, including amino, phenolic, and mercapto groups, leading to alkylation, which causes reduced bioavailability of cellular proteins and prevents the digestion and absorption of nutrients in insects and microorganisms [23]. (2) Direct toxicity of phenolic oxidation production. PPO catalyzes the generation of quinone from phenols, and the redox of quinone generates ROS, causing oxidative stress with a bactericidal effect. The production of large amounts of oxidative products results in aging, disease, and death in organisms [104]. (3) The cross-linking and polymerization of quinones with proteins or other phenols to produce melanin around injured tissue to generate a physical barrier [23,104].

There have been many studies on the response of PPO to biotic stresses, and it has been identified that PPO is one of the important enzymes involved in plant immunity. But in fact, there are still many issues that need to be discussed, such as a deeper understanding of the toxic effects or oxidative stress of PPO in organisms.

### 6.2. Response to Abiotic Stresses

Plants are often exposed to unsuitable natural environments. PPOs are involved in coping with abiotic stresses such as salt stress, drought, heavy metals, UV light, etc. PPO and a variety of related enzymes are involved in complex processes that respond to adverse environments by affecting endogenous physiological responses and altering plant traits. Thipyapong et al. found that tomato showed increased drought resistance with a lower expression of PPO [105]. Photochemical loss, photoinhibition, and photooxidation damage were reduced in plants with lower PPO under drought conditions. Overexpressed *ZmLAC1*, a laccase-related gene, could enhance maize’s ability to cope with the high salt environment, which demonstrated that laccase plays a role in the response to salt stress [106]. Heavy metals usually cause severe damage to plants, and plant PPO activity is elevated in response to heavy metal stress [107]. However, there are fewer studies in this area. By silencing the PPO gene in Clematis terniflora DC., Chen (2019) illustrated that the expression of photosynthesis-related proteins was up-regulated in plants under stress conditions [108]. It was suggested that PPO could regulate photosynthesis under stressful conditions. Szymborska-Sandhu et al. reported higher PPO activity in unshaded plants than that in shaded plants by shading assay in bastard balm (*Melittis melissophyllum* L.), which further supplied a relationship between PPO and photosynthesis [109]. Previous researches have suggested stresses response are complex progresses rather than a single enzyme or substance in plants. Therefore, the role and mechanism of PPO in the stress response should be further understood. By exploring the mechanisms involved in the response of PPO to abiotic stresses, we can help develop highly resistant cash crops using modern breeding techniques.

### 6.3. Role in Physiological Metabolism

In addition to being involved in biotic and abiotic stress, PPO is closely associated with the synthesis and degradation of metabolites in plants. It had been verified that the expressions of PPOs were specifically in *Phytolacca americana* ripe fruits during accumulation red beet pigment using northern blot assay. Based on this study, it is hypothesized that PPO may be involved in the biosynthesis of betaine [110]. In addition, it has been demonstrated that hesperidin is also synthesized by PPO [111]. Lignin, a PPO-associated metabolite, is rich in aromatic biopolymers, which play important roles in industry [112,113]. Aureusidin synthase is a copper-containing glycoprotein that belongs to the PPO family, which could catalyze the formation of aurone from chalcone to regulate flower color [114]. It had been reported that the Mehler reaction, photosynthetic priming reaction, and regulation of oxygen levels in plastids are also associated with PPO [27]. Extracellular PPO molecules are involved in the degradation of metabolites in a small number of cases. For example, extracellular polyphenol oxidase produced by fungi can degrade lignin and humus in soil [115], but it has not been reported in plants. In general, the relationship between PPO and plant metabolites is complex. PPO can both synthesize and degrade metabolites, depending on the specific phenolic compounds and environmental conditions. Hence, the role of PPO in plant metabolism requires further investigation to fully understand its complex mechanisms and potential applications in agriculture and the food industry.

## 7. Regulation of PPO Genes

PPO in plants is encoded by multiple genes. Moreover, transcript levels of PPO genes are regulated by several factors. Plants benefit from the roles of PPO in responding to stresses; therefore, studies about the expression and regulation of PPO genes are of great importance. MicroRNA (miRNA), an endogenous, nuclear-encoded, non-transcribed RNA, performs targeting identification, binding and cleaving mRNA, or blocking the translation of mRNA. miRNAs are common post-transcriptional negative regulators in the regulation of gene expression. There have been many studies demonstrating the involvement of miRNAs in the specific regulation of PPO genes in plants. The *MIR1444* genes in *Populus trichocarpa* transcript to MIR1444 that can target binding and cleavage PPOs [116,117]. Li et al. found another miRNA, smi-MIR12112, which involved in post-transcriptional regulation of PPO genes in *Salvia miltiorrhiza* [118]. Moreover, it was reported that VvMIR058 may be associated with the expression of grapevine PPOs through bioinformatic analysis [119].

Transcription factors (TFs) are essentially proteins that are involved in the regulation of gene transcription. Huang et al. identified a transcriptional activator, CsMYB59, which could regulate PPO activity by activating the expression of the *CsPPO1* gene in Camellia sinensis [96]. In *Morus notabilis*, overexpression of the MnMYB3R1 transcription factor could enhance drought resistance by enhancing the transcript of the *MnPPO1* gene [120]. Hormonal regulatory pathways in plants can also have an effect on the expression of PPO genes. Various motifs are located in the promoters of *PpPPOs,* which could respond to MeJA, salicylic acid (SA), and abscisic acid (ABA) in *Populus trichocarpa* [121]. It suggested that the expression of plant PPOs could be regulated by different hormonal pathways. Furthermore, PPO expression is critical in phytogenic food materials that are susceptible to enzymatic browning. For example, *StPOT32* gene acts as the major contributor in potato tubers [5]. The enzymic browning was inhibited significantly through suppressing *StPOT32* expression [6]. However, its regulatory mechanism is not clear. Yeast one-hybrid (Y1H) library screening is one of the common methods used in molecular biology to reveal mechanisms of gene transcription regulation. Thus, the promoter sequence of *StPOT32* gene could be cloned into Y1H vector to screen generated potato Y1H library to identify the upstream of *StPOT32* gene. Protein-protein interaction is the prerequisite for post-translational modification (PTM), which also plays a key role in regulating gene expression. Another molecular biological assay, immunoprecipitation-mass spectrometry (IP-MS), could be utilized to screen StPOT32-interaction proteins for revealing the regulatory mechanism of the *StPOT32* gene. With important physiological function of PPO, the researches about the regulation of PPO genes expression need to be further concerned in the future.

## 8. Conclusions

PPO is a copper-containing enzyme widely found in eukaryotic organisms. Its activity is dependent on pH, temperature, and phenolic substrates. Stresses can trigger the regulation of PPO activity by inducing gene expression or activating the latent PPO in plants. PPO in plants plays an important role in defense against biotic and abiotic stresses and is involved in the synthesis of many biologically active substances. Obviously, a full understanding of the regulatory mechanism of PPO activity and its mechanism of action in the process of plant stress resistance is of great interest for stress-resistant plant breeding.

The properties of PPO should be studied not only for their research value in the academic field but also for their application value in the fruit and vegetable industry. The enzymatic browning involved in PPO causes great loss, so people hope to protect the fruits and vegetables from browning in processing and storage safely and efficiently by various methods. Natural and endogenous substances that inhibit browning have received a lot of attention with a promising future. In this manuscript, we summarize the literature related to the studies of polyphenol oxidase in recent years and insert some of our reflections, which will be helpful for future research.

## Figures and Tables

**Table 1:** Optimum pH of PPO in different plants.

Species		Substrate	Optimal pH	Reference
African bush mango (*Irvingia gabonensis*) fruit peel		catechol	7.0	[48]
Tomato (*Solanum lycopersicum*)		catechol	7.0	[49]
Sweet potato (*Ipomoea batatas* L. Lam)		catechol	7.0	[16]
Lily	*Lilium lancifolium* Thunb	catechol	4.0	[50]
	*Lilium brownie* var. *viridulum*		4.0	
	*Lilium davidii* var. *unicolor*		6.5–7.0	
Fennel (*Foeniculum vulgare* Mill.) seeds		catechol	6.0	[51]
		4-methylcatechol	5.0	
		4-tertbutylcatechol	5.0	
		pyrogallol	7.0	
Truffles (*Terfezia arenaria*)		4-methylcatechol	4.0	[46]
		L-tyrosine	6.0	
		pyrogallol	6.5	
		catechol	7.0	
Soursop (*Annona muricata* L.)		catechol	6.5	[52]
Water yam (*Dioscorea alata*)		catechol	6.0	[53]
Areca nut (*Areca catechu* L) kernel		catechol	7.0	[47]
Tea leaf (*Camellia sinensis*)		catechol	PPO1: 5.5 PPO2: 6.0	[54]
Apricot (*Prunus armeniaca* L.)		catechol	4.5	[55]
Blueberry (*Vaccinium corymbosum* L.)		catechol	6.1–6.3	[56]
Mexican Golden Delicious apple (*Malus domestica*)		catechol	6.0	[57]
Elephant foot yam (Amorphophallus paeoniifolius)		catechol	6.0	[58]
Plums (*Prunus domestica*)		catechol	6.0	[59]
Taro (*Colocasia esculenta* L.)		catechol	6.8	[60]
Snow pear (*Pyrus nivalis*)		catechol	4.5	[61]
Kirmizi Kismis grape (*Vitis vinifera* L.)		4-methylcatechol	5.0	[62]

**Table 2:** Optimum temperature of PPO in different plants.

Species	Substrate	Optimal Temperature (°C)	Reference
African bush mango (*Irvingia gabonensis*) fruit peel	catechol	50	[48]
Tomato (*Solanum lycopersicum*)	catechol	50	[49]
Soursop (*Annona muricata* L.)	catechol	25	[52]
Water yam (*Dioscorea alata*)	catechol	35	[53]
Sweet potato (*Ipomoea batatas* L. Lam)	catechol	20–30	[16]
Fennel (*Foeniculum vulgare* Mill.) seeds	catechol, 4-methylcatechol, 4-tertbutylcatechol, and pyrogallol	30	[51]
Lily (*Lilium lancifolium* Thunb, *Lilium brownie* var. *viridulum, Lilium davidii* var. *unicolor*) *cotton*	catechol	15	[50]
Truffles (*Terfezia arenaria*)	4-methylcatechol	30	[46]
	L-tyrosine	35	
	pyrogallol	40	
	catechol	45	
Areca nut (*Areca catechu* L.) kernel	catechol	20	[47]
Tea leaf (*Camellia sinensis*)	catechol	PPO1: 33 PPO2: 38	[52]
Apricot (*Prunus armeniaca* L.)	catechol	45	[55]
Blueberry (*Vaccinium corymbosum* L.)	catechol	35	[56]
Mexican Golden Delicious apple (*Malus domestica*)	catechol	35	[57]
Elephant foot yam (*Amorphophallus paeoniifolius*)	catechol	35	[58]
Plums (*Prunus domestica*)	catechol	25	[59]
Taro (*Colocasia esculenta* L.)	catechol	30	[60]
Snow pear (*Pyrus nivalis*)	catechol	30	[61]
Kirmizi Kismis grape (*Vitis vinifera* L.)	4-methylcatechol	30	[62]

**Table 3:** Substrate specificity of PPO from different plant sources.

Substrate	Relative activities (%)				
	African Bush Mango (*Irvingia Gabonensis*)	Elephant Foot Yam (*Amorphophallus* *Paeoniifolius*)	Pomegranate Arils (*Punica Granatum* L. cv. Wonderful)	Mexican Golden Delicious Apple (*Malus Domestica*)	Blueberry (*Vaccinium Corymbosum* L.)
Monophenol					
Tyrosine	50.2 ± 3.34	below 1%	84.72		
Vanillin	53 ± 1.44				
Diphenol					
Catechol	100 ± 0	100	100	100	100
4-methylcatechol			200.06	354.78	26.14 ± 0.69
Caffeic acid	91 ± 8.03			14.79	
Catechin	77.1 ± 4.65				
L-DOPA	82.6 ± 6.40			54.39	16.59 ± 0.41
Chlorogenic acid		51.55		156.93	
resorcinol			35.69		2.08 ± 0.16
Trihydroxyphenol					
Gallic acid	78.6 ± 4.74	5.78	176.90		
Pyrogallol	60.5 ± 4.42	5.36	296.45	150.43	7.55 ± 0.34
Reference	[48]	[58]	[67]	[57]	[56]

**Table 4:** Molecular weight of PPO in different plants.

Enzyme Source	Molecular Weight (kDa)	Reference
African bush mango (*Irvingia gabonensis*) fruit peel	53	[48]
Fennel (*Foeniculum vulgare* Mill) seeds	27.8	[51]
Kirmizi Kismis grape (*Vitis vinifera* L.)	38.1	[62]
Kudzu (*Pueraria lobata*)	21	[69]
Truffles (*Terfezia arenaria*)	67	[46]
Water yam (*Dioscorea alata*)	32	[53]
Areca nut (*Areca catechu* L.) kernel	29.2	[47]
Tea leaf (*Camellia sinensis*)	PPO1:85 PPO2:42	[54]
Apricot (*Prunus armeniaca* L.)	37.5	[55]
Mexican Golden Delicious apple (*Malus domestica*)	58	[57]
elephant foot yam (*Amorphophallus paeoniifolius*)	40	[58]
Plums (*Prunus domestica*)	65	[59]
Taro (*Colocasia esculenta* L.)	24	[60]
Potato (*Solanum tuberosum* L.)	50	[70]

## Data Availability

Not applicable.
